# Health-related quality of life with enzalutamide versus flutamide in castration-resistant prostate cancer from the AFTERCAB study

**DOI:** 10.1007/s10147-022-02221-w

**Published:** 2022-08-10

**Authors:** Hiroji Uemura, Kazuki Kobayashi, Akira Yokomizo, Shiro Hinotsu, Shigeo Horie, Yoshiyuki Kakehi, Norio Nonomura, Osamu Ogawa, Mototsugu Oya, Kazuhiro Suzuki, Atsushi Saito, Keiko Asakawa, Satoshi Uno, Seiji Naito

**Affiliations:** 1 Department of Urology and Renal Transplantation, Yokohama City University Medical Center, 4-57 Urafune-cho, Minami-ku, Yokohama, 232-0024 Japan; 2 Department of Urology, Yokosuka Kyosai Hospital, Yonegahamadori, Yokosuka, Kanagawa 238-8558 Japan; 3 Department of Urology, Harasanshin Hospital, 1-8 Taihakumachi, Hakata-ku, Fukuoka, 812-0033 Japan; 4 Department of Biostatistics and Data Management, Sapporo Medical University, 17 Chome Minami 1 Jonishi, Chuo Ward, Sapporo, Hokkaido 060-8556 Japan; 5 Department of Urology, Juntendo University, 2 Chome-1-1 Hongo, Bunkyo City, Tokyo 113-8421 Japan; 6 Department of Medicine, Kagawa University, 1-1 Saiwaicho, Takamatsu, Kagawa 760-0016 Japan; 7 Department of Urology, Osaka University Graduate School of Medicine, 2-2 Yamadaoka, Suita, 565-0871 Japan; 8 Department of Urology, Kyoto University, Yoshidahonmachi, Sakyo Ward, Kyoto, 606-8501 Japan; 9 Department of Urology, Keio University, 2 Chome-15-45 Mita, Minato City, Tokyo 108-8345 Japan; 10 Department of Urology, Gunma University, Aramakimachi, Maebashi, Gunma 371-8510 Japan; 11 Medical Affairs Japan, Astellas Pharma Inc., 2-5-1 Nihonbashi-Honcho, Chuo-ku, Tokyo 103-8411 Japan; 12 Data Science, Development, Astellas Pharma Inc., 2-5-1 Nihonbashi-Honcho, Chuo-ku, Tokyo 103-8411 Japan

**Keywords:** HRQoL, CRPC in Japan, Patient-reported outcome, Androgen deprivation therapy, Combined androgen blockade

## Abstract

**Background:**

Patient-reported outcome (PRO) measures can provide valuable information in evaluating patients’ health-related quality of life (HRQoL). Post hoc analysis of the AFTERCAB study was conducted to evaluate the HRQoL benefit of enzalutamide plus androgen deprivation therapy (ADT) compared to flutamide plus ADT for the treatment of patients with castration-resistant prostate cancer (CRPC) in Japan.

**Methods:**

The open-label AFTERCAB study was conducted from November 2016 to March 2020 in Japanese men aged ≥ 20 years with asymptomatic or mildly symptomatic CRPC. Patients received enzalutamide plus ADT or flutamide plus ADT, respectively, as first-line alternative androgen therapy (AAT). HRQoL was analyzed through the Functional Assessment of Cancer Therapy–Prostate, EuroQoL 5-Dimension 5-Level instruments, Brief Pain Inventory–Short Form, and Brief Fatigue Inventory. The longitudinal changes in HRQoL, HRQoL deterioration based on minimally important difference (MID), and time to HRQoL deterioration were evaluated for first-line AAT.

**Results:**

Overall, HRQoL between the enzalutamide and flutamide groups was similar during first-line treatment. No statistically significant HRQoL difference in change from baseline to week 61 (least square mean difference; *p* value) was observed. Furthermore, proportions of pain progression, symptom worsening, and HRQoL deterioration based on MID, were not significantly different between groups.

**Conclusions:**

The results were similar in all subscales of each PRO, demonstrating similar HRQoL deterioration based on MID criteria between the enzalutamide and flutamide groups.

**Supplementary Information:**

The online version contains supplementary material available at 10.1007/s10147-022-02221-w.

## Introduction

According to GLOBOCAN 2020, prostate cancer (PC) is the second most frequently diagnosed cancer and fifth leading cause of cancer-related death among men, with an estimated 1.4 million new cases [1]. In Japan, PC was reported as the most common cancer in men in 2021, with an incidence of 95,400 persons per year, and the sixth most common cause of cancer-related deaths [2]. Androgen deprivation therapy (ADT) via medical or surgical castration using luteinizing hormone-releasing hormone agonists/antagonists (LHRHas) with or without antiandrogen drugs has remained the mainstay of treatment for PC for decades [3, 4]. Combined androgen blockade (CAB) therapy of bicalutamide and ADT is commonly used to treat PC in Japan [5–7]. Though ADT offers near certain remission, cancer cells become resistant to ADT, leading to disease reactivation and transition to a lethal phenotype—castration-resistant PC (CRPC) [8]. The Japanese Urological Association’s 2012 PC guidelines recommend alternative androgen therapy (AAT) with flutamide plus ADT for the treatment of patients with CRPC who progress despite CAB [9]. Recently developed new agents, including hormonal therapy, such as enzalutamide, have significantly transformed CRPC management, increasing overall survival and quality of life (QoL) [4].

Enzalutamide is a targeted oral androgen receptor (AR) inhibitor that binds competitively to the ligand-binding domain of the AR, thus inhibiting nuclear translocation of the AR, DNA binding, and coactivator recruitment [10]. Its clinical activities have been well established in phase 3 randomized clinical trials [11–15]. Japan’s Ministry of Health, Labour and Welfare approved enzalutamide for the treatment of men with CRPC in 2014 [16] and recently amended the indication to include metastatic hormone-sensitive PC and PC with distant metastasis, based on the ARCHES (NCT02677896) [15] and ENZAMET (NCT02446405) trials [17].

With the evolving therapeutic landscape and the need for long-term therapy, the potential impact of adverse effects on patients’ health-related QoL (HRQoL) is a pressing challenge for clinicians treating PC [18]. Cancer-related fatigue, a multifaceted concept comprising the physical, social, emotional, and psychological symptoms that patients undergoing cancer treatment experience, is commonly reported among men with PC [19–22]. The Erim et al. study reported that PC-related anxiety had notable associations with low mood/nervousness, productivity loss, and risk of probable depression, impacting patients’ HRQoL [23]. Novel hormonal therapy has a different spectrum of adverse events (AEs) that may impact HRQoL [14]. In phase 3 trials, AEs such as hot flash, fatigue, arthralgia, hypertension, increased weight, and diarrhea were observed with enzalutamide; however, it achieved a favorable safety profile overall, with no unexpected AEs and fewer reported ≥ grade 3 AEs [15]. Although the oncological benefits for a plethora of new agents have been studied, the associated HRQoL evidence is scarce. HRQoL for patients with PC comprises specific urological-related symptoms and overall HRQoL. The currently used validated questionnaires to measure HRQoL for a patient with PC include, but are not limited to, the Functional Assessment of Cancer Therapy–Prostate (FACT-P) [24], EuroQoL 5-Dimension 5-Level instruments (EQ-5D-5L) [25], Brief Pain Inventory–Short Form (BPI-SF) [26], and Brief Fatigue Inventory (BFI) [27].

A previous publication from the AFTERCAB (NCT02918968) study conducted in Japanese patients with CRPC who failed CAB therapy with bicalutamide compared the efficacy and safety of enzalutamide plus ADT and flutamide plus ADT. The time to prostate-specific antigen (PSA) progression was significantly extended by enzalutamide compared to flutamide, while both therapies had acceptable safety profiles [9]. This post hoc analysis of the AFTERCAB study determined the HRQoL benefit of enzalutamide plus ADT therapy compared to flutamide plus ADT for the treatment of patients with CRPC. The change in HRQoL was assessed through four patient-reported outcome (PRO) measures—FACT-P, EQ-5D-5L, BFI, and BPI-SF using the existing data set of the AFTERCAB study.

## Materials and methods

### Study design

AFTERCAB was a randomized, open-label, phase 4 comparative study of enzalutamide versus flutamide between November 2016 and March 2020 in Japanese men with metastatic or nonmetastatic CRPC who relapsed during CAB therapy with bicalutamide were randomized to enzalutamide or flutamide for first-line AAT [29]. Dynamic allocation was performed as per the stages M0/N0 (no distant metastasis and no lymph node metastasis), M0/N1 (no distant metastasis, but metastasis in lymph nodes distal to the aortic bifurcation), or M1 (distant metastasis, including metastasis in lymph nodes proximal to the aortic bifurcation) through the biased coin technique. Patients received enzalutamide plus ADT or flutamide plus ADT, respectively, as first-line AAT, hereupon referred to as the enzalutamide group and flutamide group, and were switched to second-line AAT following PSA progression. The treatment period with each drug was ≤ 2 years from last patient enrollment. PSA progression was defined according to the consensus guidelines of the Prostate Cancer Clinical Trials Working Group [28].

### Study population

The study included Japanese men aged ≥ 20 years diagnosed with histologically/cytologically confirmed adenocarcinoma of the prostate without neuroendocrine differentiation or small-cell histology on continuous ADT with gonadotropin-releasing hormone agonist/antagonist or bilateral orchiectomy. Study participants must have had asymptomatic or mildly symptomatic CRPC with disease progression despite CAB therapy, or bicalutamide and ADT. Study inclusion and exclusion criteria are described in Online Resource Table 1. All study participants signed an informed consent form approved by the institutional review board at each study center. The study participants received enzalutamide 160 mg/day or flutamide 375 mg/day (125 mg three times daily), as instructed on the package inserts.

### Study endpoints

The study evaluated the PROs at week 1 (day 1), week 13, every subsequent 12 weeks, and at completion/discontinuation for first-line and second-line AAT, based on information reported by the patients. HRQoL was analyzed by the FACT-P, EQ-5D-5L, BPI-SF, and BFI. In these post hoc analyses, the longitudinal changes in HRQoL, HRQoL deterioration based on minimally important difference (MID), and time to HRQoL deterioration were evaluated for first-line AAT. The threshold for MID from baseline as pain progression, symptom worsening, and HRQoL deterioration are defined in Online Resource Table 2.

### FACT-P

FACT-P is a PC-specific multifaceted QoL scale. It includes 27 core items to evaluate patients’ functions in four areas: physical health, social/family, emotional, and functional well-being. Twelve site-specific items were added to assess prostate-related symptoms via a 5-grade Likert scale. The aggregate of all items was the overall QoL score (ranging from 0 to 156, with a higher score indicating better QoL).

### EQ-5D-5L

The EQ-5D-5L is a generic, preference-based measure comprising five items (mobility, self-care, usual activities, pain/discomfort, and anxiety/depression), with each item evaluated at five levels, from “no problem” to “extreme problem.” The overall score ranges from – 0.025 (a state worse than dead) to 1 (perfect health), with 0 as the state of being dead. The last question was a visual analog scale (VAS) to evaluate the present health status in the range from “best imaginable health status (score of 100)” to “worst imaginable health status (score of 0).” For EQ-5D-5L, the higher the score, the better the QoL.

### BPI-SF

The BPI is a questionnaire slip verified as a self-assessment scale measuring a patient’s level of pain, the effect of pain on activities of daily living, and analgesic use. The study used the BPI-SF scale comprising questions in nine categories for which numerical scales from 0 (best) to 10 (worst) were used. The investigator/subinvestigator instructed patients to describe their PC-related pain. For the BPI-SF, the lower the score, the better the QoL.

### BFI

The BFI is a questionnaire comprising questions in 10 categories to evaluate the malaise (subjective symptoms characterized as debility, which includes physical and mental wasting) of cancer patients. For the BFI, the overall score ranges from 0 to 10 (the lower the score, the better the QoL).

### Statistical analysis

All analyses were done in the intent-to-treat (ITT) population, defined as all patients randomized. The data analyses were performed using SAS, version 9.4 (SAS Institute, Cary, NC, USA). All statistical comparisons were conducted using two-sided tests at the 5% significance level. As a general principle, no imputation of missing data was done unless specified otherwise. For change in HRQoL, mixed model repeated measure (MMRM) analysis was conducted for change from baseline in each endpoint to postbaseline visits, using the baseline value and status of distant metastasis as covariates, analysis visits (categorical variable), and interaction of [treatment group x visit] as fixed effects. The analysis window for MMRM was until week 61 in first-line AAT to remove biases introduced by high patient dropout beyond this time point. The primary hypothesis tested the difference between least squares mean (LSM) change from baseline to week 61. The pattern mixture models (PMM) with delta-adjusted multiple imputation (MI) were conducted under missing-not-at-random assumption.

The frequency and proportion of patients who achieved HRQoL deterioration were evaluated. The stratified Cochran–Mantel–Haenszel test was used to compare the proportion of MID between groups, with stratification factor as the disease stages (M0/N0, M0/N1, or M1). For the time to HRQoL deterioration, benefit of enzalutamide compared to flutamide was assessed with the stratified log-rank test. Unstratified Cox proportional hazards model with treatment group and disease stage as the covariate was used to support the log-rank test. Kaplan–Meier (KM) curves were used to estimate the distribution of duration of event-free survival. The 50th percentile of KM estimates was used to estimate the median duration of event.

## Results

Overall, 253 patients were enrolled in the study, 47 of whom discontinued prior to randomization. The remaining 206 patients were randomized to an enzalutamide (*n* = 102) or flutamide (*n* = 104) group.

### Baseline patient characteristics

#### Baseline demographics

Median age was 76.0 years in the enzalutamide group and 74.5 years in the flutamide group, with a similar proportion of patients in each age category. The baseline characteristics are provided in (Table 1). In both treatment groups, the disease stage at randomization was M1 (enzalutamide group: 73.5%; flutamide group: 72.1%) or M0/N0 (23.5%/24.0%, respectively) for most patients.

**Table 1:** Patient baseline characteristics: demographics and disease history (ITT population)

Parameter statistic	Enzalutamide for first AAT group (*n* = 102)	Flutamide for first AAT group (*n* = 104)	Total (*n* = 206)
Age, years			
Mean	74.4	74.1	74.2
Age category, years (%)			
< 65	10 (9.8)	11 (10.6)	21 (10.2)
65–74	35 (34.3)	41 (39.4)	76 (36.9)
75–84	47 (46.1)	43 (41.3)	90 (43.7)
≥ 85	10 (9.8)	9 (8.7)	19 (9.2)
Weight, kg			
Mean (SD)	67.65 (10.13)	66.90 (9.89)	67.27 (9.99)
BMI, kg/m^2^			
Mean (SD)	25.20 (3.29)	24.83 (2.80)	25.01 (3.05)
Baseline serum PSA value, ng/mL			
Mean (SD)	37.03 (92.67)	32.26 (84.57)	34.62 (88.49)
Baseline LDH value, U/L			
Mean (SD)	209.3 (58.5)	196.6 (49.6)	202.9 (54.5)
Baseline hemoglobin value, g/dL			
Mean (SD)	12.98 (1.30)	13.33 (1.24)	13.16 (1.28)
Baseline ALP value, U/L			
Mean (SD)	314.8 (297.6)	300.8 (237.8)	307.7 (268.5)
Baseline serum albumin value, g/dL			
Mean (SD)	4.3 (0.3)	4.4 (0.3)	4.3 (0.3)
Baseline creatinine value, mg/dL			
Mean (SD)	0.88 (0.24)	0.92 (0.25)	0.90 (0.25)
Baseline ECOG performance status, *n* (%)			
0	91 (89.2)	90 (86.5)	181 (87.9)
1	11 (10.8)	14 (13.5)	25 (12.1)
Baseline BPI-SF question 3, *n* (%)			
0–1	92 (90.2)	82 (78.8)	174 (84.5)
2–3	10 (9.8)	22 (21.2)	32 (15.5)
> 3	0	0	0
History of prior cardiovascular disease, *n* (%)			
Yes	82 (80.4)	73 (70.2)	155 (75.2)
No	20 (19.6)	31 (29.8)	51 (24.8)
Disease stages at randomization, *n* (%)			
M0/N0	24 (23.5)	25 (24.0)	49 (23.8)
M0/N1	3 (2.9)	4 (3.8)	7 (3.4)
M1	75 (73.5)	75 (72.1)	150 (72.8)
Regional lymph nodes at randomization, *n* (%)			
NX	0	0	0
N0	75 (73.5)	81 (77.9)	156 (75.7)
N1	27 (26.5)	23 (22.1)	50 (24.3)
Distant metastasis at randomization, *n* (%)			
MX	0	0	0
M0	27 (26.5)	29 (27.9)	56 (27.2)
M1	75 (73.5)	75 (72.1)	150 (72.8)
Time from initial diagnosis (months)			
* n*	101	104	205
Mean	42.35 (42.95)	40.29 (37.52)	41.31 (40.20)
Total Gleason score at initial diagnosis			
* n*	98	103	201
Mean (SD)	8.5 (0.9)	8.4 (0.9)	8.5 (0.9)
Primary + secondary Gleason scores, *n* (%)			
< 3 + 3	0	0	0
3 + 3	2 (2.0)	3 (2.9)	5 (2.4)
3 + 4	2 (2.0)	6 (5.8)	8 (3.9)
4 + 3	9 (8.8)	10 (9.6)	19 (9.2)
4 + 4	24 (23.5)	23 (22.1)	47 (22.8)
4 + 5	36 (35.3)	35 (33.7)	71 (34.5)
5 + 4	14 (13.7)	21 (20.2)	35 (17.0)
5 + 5	7 (6.9)	4 (3.8)	11 (5.3)
Other	3 (2.9)	0	3 (1.5)
Missing	5 (4.9%)	2 (1.9%)	7 (3.4%)
Total Gleason score category at initial diagnosis, *n* (%)			
Low (2–4)	0	0	0
Medium (5–7)	14 (13.7)	19 (18.3)	33 (16.0)
High (8–10)	84 (82.4)	84 (80.8)	168 (81.6)
Missing	4 (3.9)	1 (1.0)	5 (2.4)
Clinical tumor stage at initial diagnosis, *n* (%)			
TX	1 (1.0)	0	1 (0.5)
T0	0	0	0
T1	8 (7.8)	7 (6.7)	15 (7.3)
T2	14 (13.7)	16 (15.4)	30 (14.6)
T3	51 (50.0)	50 (48.1)	101 (49.0)
T4	26 (25.5)	31 (29.8)	57 (27.7)
Unknown	2 (2.0)	0	2 (1.0)
Regional lymph nodes at initial diagnosis, *n* (%)			
NX	0	0	0
N0	56 (54.9)	46 (44.2)	102 (49.5)
N1	43 (42.2)	58 (55.8)	101 (49.0)
Unknown	3 (2.9)	0	3 (1.5)
Distant metastasis at initial diagnosis, *n* (%)			
MX	0	0	0
M0	40 (39.2)	36 (34.6)	76 (36.9)
M1	59 (57.8)	68 (65.4)	127 (61.7)
Unknown	3 (2.9)	0	3 (1.5)
PSA doubling time, months			
* n*	102	104	206
Mean (SD)	4.35 (8.30)	4.23 (6.74)	4.29 (7.54)
PSA progression at study entry, *n* (%)			
Yes	101 (99.0)	104 (100.0)	205 (99.5)
No	1 (1.0)	0	1 (0.5)
Type of disease progression at study entry, *n* (%)			
PSA progression only	100 (98.0)	104 (100.0)	204 (99.0)
Radiographic progression with/without PSA progression	2 (2.0)	0	2 (1.0)
Disease localization at screening, *n* (%)			
Bone only	0	0	0
Soft tissue only	36 (35.3)	37 (35.6)	73 (35.4)
Bone and soft tissue	66 (64.7)	67 (64.4)	133 (64.6)
None	0	0	0
Target or nontarget soft tissue disease at screening, *n* (%)			
Yes	43 (42.2)	49 (47.1)	92 (44.7)
No	59 (57.8)	55 (52.9)	114 (55.3)
Extent of disease at screening, *n* (%)			
Bone	0	0	0
Lymph node	23 (22.5)	19 (18.3)	42 (20.4)
Visceral lung	7 (6.9)	5 (4.8)	12 (5.8)
Visceral liver	3 (2.9)	2 (1.9)	5 (2.4)
Visceral lung and/or liver	9 (8.8)	6 (5.8)	15 (7.3)
Other soft tissue	17 (16.7)	29 (27.9)	46 (22.3)

*AAT* alternative antiandrogen therapy, *ALP* alkaline phosphatase, *BMI* body mass index, *BPI-SF* Brief Pain Inventory–Short Form, *ECOG* Eastern Cooperative Oncology Group, *ITT* intent-to-treat, *LDH* lactate dehydrogenase, *M0* no distant metastasis, *M1* distant metastasis, *MX* distant metastasis cannot be assessed, *N0* no lymph node metastasis, *N1* metastasis in lymph nodes distal to aortic bifurcation, *NX* regional lymph nodes cannot be assessed, *PSA* prostate-specific antigen, *SD* standard deviation

#### Baseline disease history

Median time from initial diagnosis of PC was 26.02 months in the enzalutamide group and 25.92 months in the flutamide group. Approximately 80% of patients in both groups had a Gleason score of 8–10 at the time of initial diagnosis. More than 60% of patients in both groups entered the study with both bone and soft tissue metastases. Baseline disease characteristics were generally similar across the two treatment groups.

### PRO assessment

#### FACT-P

**Fig. 1:**
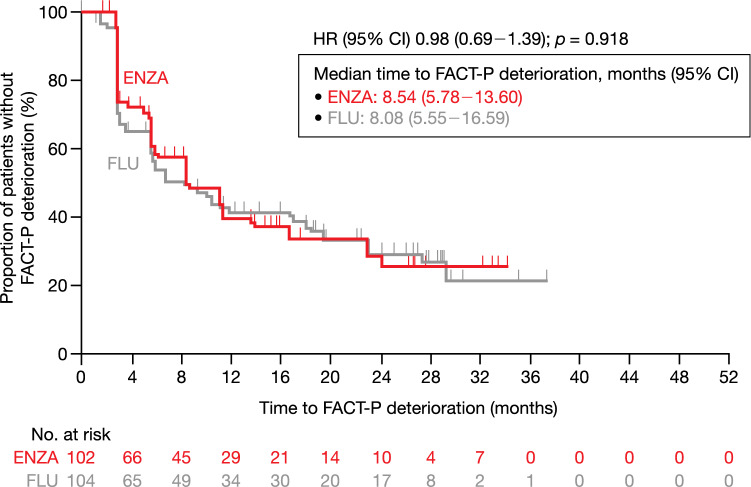
Time to FACT-P total score deterioration based on MID (first-line). HR: the benefit of ENZA compared to FLU was evaluated by HR (ENZA/FLU), with its 95% CI based on Cox proportional hazards model. *p* value was calculated on a log-rank test stratified with disease stages. *CI* confidence interval, *ENZA* enzalutamide, *FACT-P* Functional Assessment of Cancer Therapy–Prostate, *FLU* flutamide, *HR* hazard ratio, *MID* minimally important difference

The LSM changes from baseline to week 61 in FACT-P score to first-line AAT were – 6.7 in the enzalutamide group and – 1.2 in the flutamide group. The mean difference (95% confidence interval [CI]) between the enzalutamide group and flutamide group was – 5.5 (– 12.7 to 1.7), which was not statistically significant (*p* = 0.135). The median time to FACT-P deterioration (95% CI) was 8.54 months (5.78 to 13.60) for the enzalutamide group and 8.08 months (5.55 to 16.59) for the flutamide group (Fig. 1). The proportion of symptom worsening as MID in FACT-P score to first-line AAT was not significantly lower in the enzalutamide group (60.6% [60/99]) than in the flutamide group (65.4% [68/104]) [*p* = 0.490]. The MMRM analysis for the change in FACT-P score is demonstrated in Online Resource Fig. 1.

#### EQ-5D-5L

The LSM changes from baseline to week 61 in the EQ-5D-5L utility index to first-line AAT were – 0.0426 in the enzalutamide group and – 0.0495 in the flutamide group. The mean difference (95% CI) between the enzalutamide and flutamide group was 0.0070 (– 0.0579 to 0.0718), which was not statistically significant (*p* = 0.831). The LSM changes from baseline to week 61 in EQ-5D-5L VAS to first-line AAT were – 1.7 in the enzalutamide group and – 3.8 in the flutamide group. The mean difference (95% CI) between the enzalutamide group and flutamide group was 2.1 (– 3.5 to 7.7), which was not statistically significant (*p* = 0.457). The proportion of symptom worsening as MID in EQ-5D-5L utility index to the first-line AAT was not significantly lower in the enzalutamide group (41.4% [41/99]) than in the flutamide group (44.2% [46/104]) [*p* = 0.698]. For EQ-5D-5L VAS, MID to the first-line AAT was not significantly lower in the enzalutamide group (58.6% [58/99]) than in the flutamide group (69.2% [72/104]) [*p* = 0.121].

**Fig. 2:**
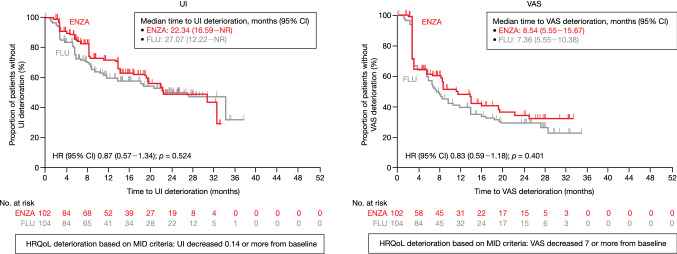
Time to EQ-5D-5L deterioration based on MID (first-line). HR: the benefit of enzalutamide compared to flutamide was evaluated by HR (ENZA/FLU), with its 95% CI based on Cox proportional hazards model. *p* value was calculated on a log-rank test stratified with disease stages. *CI* confidence interval, *ENZA* enzalutamide, *EQ-5D-5L* EuroQol 5-Dimension 5-Level instruments, *FLU* flutamide, *HR* hazard ratio, *HRQoL* health-related quality of life, *MID* minimally important difference, *NR* not reached, *UI* utility index, *VAS* visual analog scale

The KM curve and MMRM analysis for the EQ-5D-5L utility index and EQ-5D-5L VAS are demonstrated in Fig. 2 and Online Resource Fig. 2, respectively.

#### BPI-SF

The LSM changes from baseline to week 61 in the BPI-SF pain severity score to first-line AAT were 0.1 in the enzalutamide group and 0.2 in the flutamide group. The mean difference (95% CI) between the enzalutamide group and flutamide group was 0.0 (– 0.5 to 0.4), which was not statistically significant (*p* = 0.908). For the BPI-SF pain interference score, the LSM changes from baseline to week 61 were 0.22 in the enzalutamide group and 0.06 in the flutamide group. The mean difference (95% CI) between the enzalutamide group and flutamide group was 0.16 (– 0.23 to 0.55), which was not statistically significant (*p* = 0.419). Similarly, no statistically significant difference in change from baseline to week 61 was observed in other subscales, including worst pain, least pain, average pain, and pain now.

**Fig. 3:**
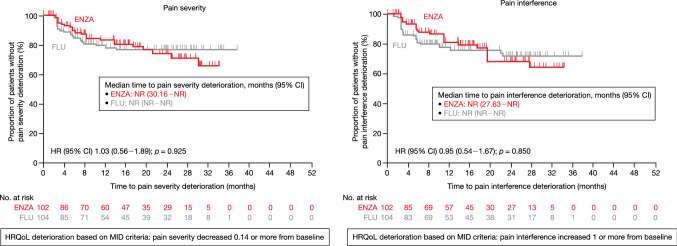
Time to BPI-SF deterioration based on MID (first-line). HR: the benefit of ENZA compared to FLU was evaluated by HR (ENZA/FLU), with its 95% CI based on Cox proportional hazards model. *p* value was calculated on a log-rank test stratified with disease stages. *BPI-SF* Brief Pain Inventory–Short Form, *CI* confidence interval, *ENZA* enzalutamide, *FLU* flutamide, *HR* hazard ratio, *HRQoL* health-related quality of life, *MID* minimally important difference, *NR* not reached

The proportion of symptom worsening as MID in BPI-SF pain severity to the first-line AAT was similar in the enzalutamide group (20.6% [21/102]) and flutamide group (20.2% [21/104]) [*p* = 0.941]. The proportion of symptom worsening as MID in BPI-SF pain interference to the first-line AAT was similar in the enzalutamide group (22.5% [23/102]) and flutamide group (24.0% [25/104]) [*p* = 0.783]. The KM curve and MMRM analysis for the BPI-SF are demonstrated in Fig. 3 and Online Resource Fig. 3, respectively.

#### BFI

**Fig. 4:**
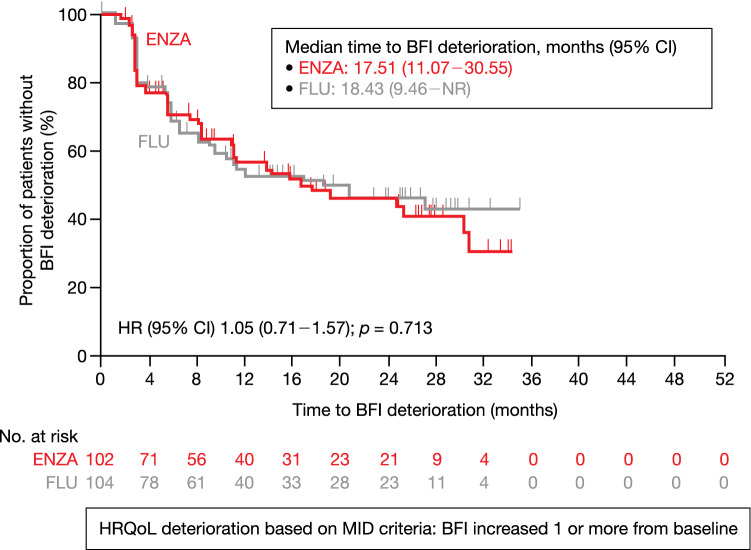
Time to BFI global score deterioration based on MID (first-line). HR and *p* value were calculated using unstratified Cox proportional hazards model with treatment and disease as covariate. *p* value was calculated on a log-rank test stratified with disease stages. *BFI* Brief Fatigue Inventory, *CI* confidence interval, *ENZA* enzalutamide, *FLU* flutamide, *HR* hazard ratio, *HRQoL* health-related quality of life, *MID* minimally important difference, *NR* not reached

The LSM changes from baseline to week 61 in the BFI score to first-line AAT were 0.50 in the enzalutamide group and 0.27 in the flutamide group. The mean difference (95% CI) between the enzalutamide group and flutamide group was 0.23 (– 0.35 to 0.81), which was not statistically significant (*p* = 0.430). The proportion of symptom worsening as MID in BFI score to the first-line AAT was similar in the enzalutamide group (48.0% [49/102]) and flutamide group (48.1% [50/104]) [*p* = 0.982]. The KM curve and MMRM analysis for the BFI are demonstrated in (Fig. 4) and Online Resource Fig. 4, respectively.

## Discussion

This post hoc analysis of the AFTERCAB study assessed the benefit of enzalutamide plus ADT compared to flutamide plus ADT assessed by PRO measures in Japanese patients with CRPC. Overall, the HRQoL between the enzalutamide and flutamide groups was not statistically different during first-line treatment among all PRO measures, longitudinal change from baseline, proportion of HRQoL deterioration, and time to HRQoL deterioration measured by the FACT-P, EQ-5D-5L, BPI-SF, and BFI global score. The plausible rationale for the similarity in HRQoL between the two treatment groups during first-line treatment may be because the recruited patients had relatively early phase CRPC with limited impact on HRQoL. In addition, since the treatment switch decision to second-line was made based on PSA progression, the general health condition of patients was maintained at the end of first-line treatment without significant deterioration in PRO.

Currently, limited evidence on QoL outcomes is available in Japanese patients with PC [29]. The Arai et al. study that assessed HRQoL in 203 Japanese patients with PC concluded that the maximum androgen blockade (MAB) with bicalutamide plus LHRHas did not reduce overall QoL, though the MAB was superior to monotherapy in achieving early improvement of QoL related to micturition disorder and pain [29]. Previous studies assessing the effect of enzalutamide on HRQoL in randomized phase 3 trials demonstrated improved HRQoL versus placebo, confirming that the addition of enzalutamide allowed patients to maintain their HRQoL [30–33]. In the Cella et al. analyses of longitudinal changes in FACT-P scores in the AFFIRM trial, mean FACT-P score decreased 1.52 points with enzalutamide compared to 13.73 points with placebo (*p* < 0.001) after 25 weeks. Significant treatment differences favoring enzalutamide were observed for all FACT-P subscales [30]. In a randomized phase 3 trial assessing HRQoL in men with nonmetastatic CRPC, enzalutamide showed a clinical benefit by delaying pain progression, symptom worsening, and decrease in functional status compared with placebo [33]. These results are consistent with this study in which enzalutamide exhibited a significantly extended time to PSA progression, with no significant change in HRQoL compared to flutamide during the treatment period. Enzalutamide may be a valuable addition to the treatment armamentarium of PC in Japan, as shown by previous study results validating its efficacy and safety. In the Iguchi et al. study, the 3 month (80.8% versus 35.3%) and 6 month (73.1% versus 31.4%) PSA response rates were significantly higher in patients receiving enzalutamide compared to flutamide, respectively [34]. These study results corroborate the benefits previously published from the AFTERCAB study from a patient’s perspective. Results from the AFTERCAB study assessing the efficacy and safety of enzalutamide also showed improvement in ≥ 50% PSA response rate with first-line therapy (72.5% for enzalutamide first versus 34.6% for flutamide first) [9].

Although this study is a preliminary analysis demonstrating the comparison of enzalutamide and flutamide by changes in HRQoL in Japanese patients with CRPC, the study results must be interpreted with caution. This open-label study did not investigate overall or cancer-specific survival. Various statistical methods are available to analyze longitudinal data and assess missing data. The MMRM method assumes that the missing data follow the pattern of patients remaining in the study. Though the analysis window for MMRM in the study was up to week 61 to remove biases introduced by missing data, the results of the MMRM analysis should be interpreted with caution due to a substantial amount of missing data, especially in the flutamide group. A PMM analysis with delta-adjusted MI showed results consistent with MMRM. Moreover, these results were derived from a post hoc analysis that was not initially designed for the comparison of PROs; hence, it was unlikely to demonstrate statistical significance. As an additional limitation of the crossover study design, the study treatment was switched to other upon PSA progression, making it difficult to interpret results. Despite these limitations, the study presents vital findings related to HRQoL outcomes that are beneficial to further inform the use of enzalutamide in Japan.

The study demonstrated that similar HRQoL deterioration based on MID criteria between the enzalutamide and flutamide groups’ changes in HRQoL were stable, and no significant change from baseline to week 61 was observed in either group. Enzalutamide, therefore, significantly extended time to PSA progression compared to flutamide, with no significant change in HRQoL during the enzalutamide treatment period.

## Supplementary Information

Below is the link to the electronic supplementary material.

Supplementary file1 (PDF 373 KB)

## Data Availability

Upon request, and subject to certain criteria, conditions, and exceptions, Astellas will provide access to anonymised patient level data from completed Astellas sponsored Phase 1 to 4 interventional clinical studies conducted for products and indications which have been approved in any country and also for studies conducted for terminated compounds. Approval must have been granted by the agencies of the main regions US, EU and Japan. If approval is sought in only one or two regions, approval must have been granted by those agencies. Where available, the following anonymised patient level data and information is provided for each clinical study: Raw dataset, Analysis ready dataset, Protocols with any amendments or addenda, Annotated case report form, Statistical analysis plan, Dataset specifications and Clinical study report. Additionally data may be available upon request. Researchers may request access to anonymized participant level data, trial level data and protocols from Astellas sponsored clinical trials at www.clinicalstudydatarequest.com. For the Astellas criteria on data sharing see: https://clinicalstudydatarequest.com/Study-Sponsors/Study-Sponsors-Astellas.aspx.
