# Supplementary material for: Health-related quality of life with enzalutamide versus flutamide in castration-resistant prostate cancer from the AFTERCAB study
Source: Int J Clin Oncol. 2022 Aug 10;27(10):1632–43. doi: 10.1007/s10147-022-02221-w (PMC9510122; doi:10.1007/s10147-022-02221-w)
Supplement: Supplementary file 1 — Supplementary file1 (PDF 373 KB) [file 10147_2022_2221_MOESM1_ESM.pdf]

Online resource

Health-related quality of life with enzalutamide versus flutamide in castration-resistant prostate cancer from the AFTERCAB study

Int J Clin Oncol

Hiroji Uemura* • Kazuki Kobayashi • Akira Yokomizo • Shiro Hinotsu • Shigeo Horie Yoshiyuki Kakehi • Norio Nonomura • Osamu Ogawa • Mototsugu Oya • Kazuhiro Suzuki • Atsushi Saito • Keiko Asakawa • Satoshi Uno • Seiji Naito

* **Corresponding author** Department of Urology and Renal Transplantation, Yokohama City University Medical Center, 4-57 Urafune-cho, Minami-ku, Yokohama 232-0024, Japan; Tel: +81-045-261-5656; Fax: +81-045-253-1962; hu0428@yokohama-cu.ac.jp

Online Resource Table 1 Study inclusion/exclusion criteria

Inclusion criteria	Exclusion criteria
Age ≥ 20 years at time of signing informed consent form	Patients with severe concurrent diseases, infections, or complications, which were considered inappropriate for enrollment by the investigator/subinvestigator
Diagnosed with histologically or cytologically confirmed adenocarcinoma of the prostate without neuroendocrine differentiation or small-cell histology	Patients with confirmed or suspected brain metastasis or active leptomeningeal metastasis
Continuous ADT with GnRH agonist/antagonist or bilateral orchiectomy (surgical or chemical castration)	Patients with a history of malignant tumor other than prostate cancer in the past 5 years (except for nonmelanoma skin cancer cured with radical therapy)
Patients for whom treatment with effective GnRH agonist/antagonist was to be continued during the study period if bilateral orchiectomy was not performed	Patients hypersensitive to the ingredients of enzalutamide capsules or flutamide tablets
Serum testosterone level ≤ 1.73 nmol/L (50 ng/dL or 0.5 ng/mL) at screening visit	Patients with history of seizure or any condition that could predispose to seizure
Patients with no change in dose of bisphosphonate preparation or denosumab for at least 4 weeks if these drugs were used	Patients with liver disorders such as viral hepatitis and hepatic cirrhosis, or patients with AST and ALT at screening visit higher than the ULN
Patients with asymptomatic or mildly symptomatic prostate cancer (BPI-SF score < 4 for question 3, “the worst pain within 24 hours”	Patients on warfarin
Patients with an ECOG performance status of 0 or 1	Patients received treatment for prostate cancer with cytocidal chemotherapy that included antiandrogenic agents other than bicalutamide (e.g., enzalutamide, flutamide), abiraterone, or estramustine

Patients with an estimated life expectancy of ≥ 12 months	Patients participated in clinical study on a drug other than GnRH agonists/antagonists in prostate cancer
Patient had progression of the disease during CAB therapy in combination with bicalutamide and ADT in the following criteria:<br>• PSA increase confirmed at least two time points with an interval of ≥ 1 week. PSA at screening visit had to be ≥ 2 ng/mL (2 μg/L)<br>• Soft tissue disease progression defined by RECIST guidelines<br>• Progression of ≥ 2 bone lesions defined as new lesions in bone scintigraphy by PCWG2	Patients received treatment with herbal medications that could have hormonal antiprostate cancer activity or herbal medications (saw palmetto) that could decrease PSA levels; patients received treatment for prostate cancer with systemic corticosteroids or treatment for other diseases with systemic corticosteroids greater than the equivalent of 10 mg per day of prednisone (dexamethasone 1 mg/day) within 4 weeks prior to enrollment (day 1)
	Patients received treatment with bicalutamide within 6 weeks prior to enrollment
	Patients received treatment with 5-α reductase inhibitors (finasteride, dutasteride), estrogens, or drugs with antitumor action other than GnRH agonists/antagonists within 4 weeks prior to enrollment (day 1)
	Patients received treatment with opioid analgesic for pains associated with prostate cancer within 4 weeks prior to enrollment (day 1)

ADT androgen deprivation therapy; *ALT* alanine aminotransferase; *AST* aspartate aminotransferase; *BPI-SF* Brief Pain Inventory–Short Form; *CAB* combined androgen blockade; *ECOG* Eastern Cooperative Oncology Group; *GnRH* gonadotropin-releasing hormone; *PCWG2* Prostate Cancer Working Group 2; *PSA* prostate-specific antigen; *RECIST* Response Evaluation Criteria in Solid Tumors; *ULN* upper limit of normal

Online Resource Table 2 Threshold for MID

	Possible score range	Established MID range	MID threshold in this study
FACT-P			
Total score	0 to 156	6 to 10	10
Prostate cancer subscale	0 to 48	2 to 3	3
Physical well-being	0 to 28	2 to 3	3
Functional well-being	0 to 28	2 to 3	3
Emotional well-being	0 to 24	2 to 3	3
Social or family well-being	0 to 28	2 to 3	3
EQ-5D-5L			
EQ-5D utility index	–0.025 to 1	0.04 to 0.14	0.14
EQ-5D VAS	0 to 100	7	7
BPI-SF			
Pain severity	0 to 10	2	2
Worst pain	0 to 10	2	2
Least pain	0 to 10	2	2
Average pain	0 to 10	2	2
Pain now	0 to 10	2	2
Pain interference	0 to 10	1 to 2	1
BFI			
Global BFI score	0 to 10	Not available	1[a]

BFI Brief Fatigue Inventory; *BPI-SF* Brief Pain Inventory–Short Form; *EQ-5D-5L* EuroQoL 5-Dimension 5-Level instruments; *FACT-P* Functional Assessment of Cancer Therapy–Prostate; *MID* minimally important difference; *SD* standard deviation

[a]No specific reference is available. Since one-half of the SD pooled over the two treatment groups at baseline was 0.42, smaller than 1, which is the minimum feasible change for global BFI score, MID threshold in this study was determined to be 1

Online Resource Fig. 1 MMRM analysis of change in FACT-P total score (first-line)

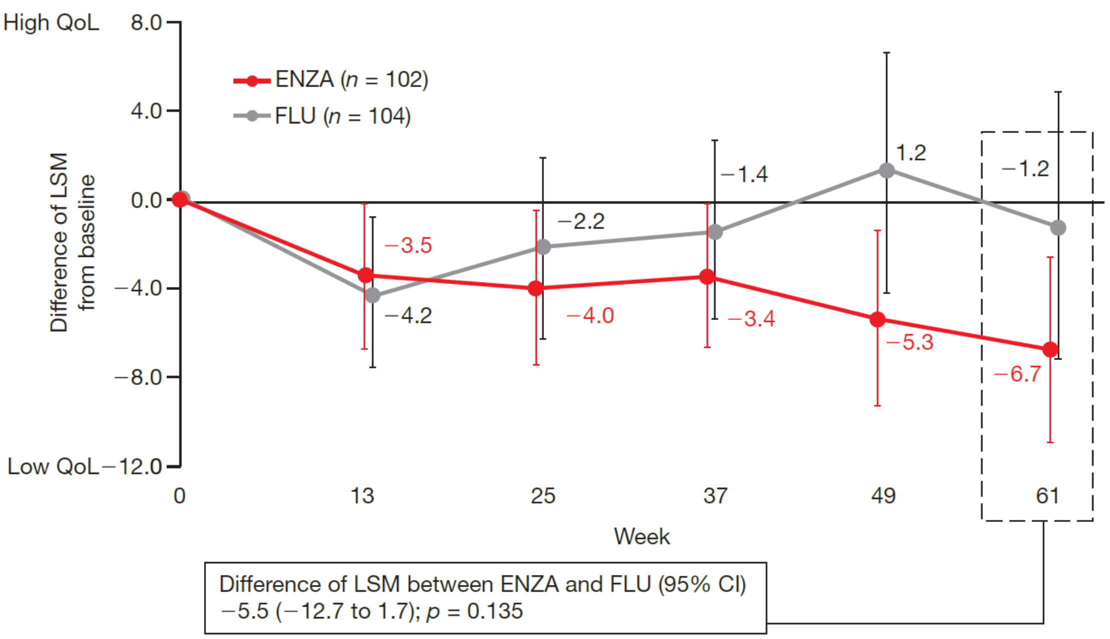

An REML approach was used. Kenward-Roger approximation was used to estimate the degree of freedom of denominator, and "unstructured" was used for structure of variance-covariance matrix

CI confidence interval; *ENZA* enzalutamide; *FACT-P* Functional Assessment of Cancer Therapy–Prostate; *FLU* flutamide; *LSM* least squares means; *MMRM* mixed model repeated measures; *QoL* quality of life; *REML* restricted maximum likelihood

Online Resource Fig. 2 Change in EQ-5D-5L (first-line)

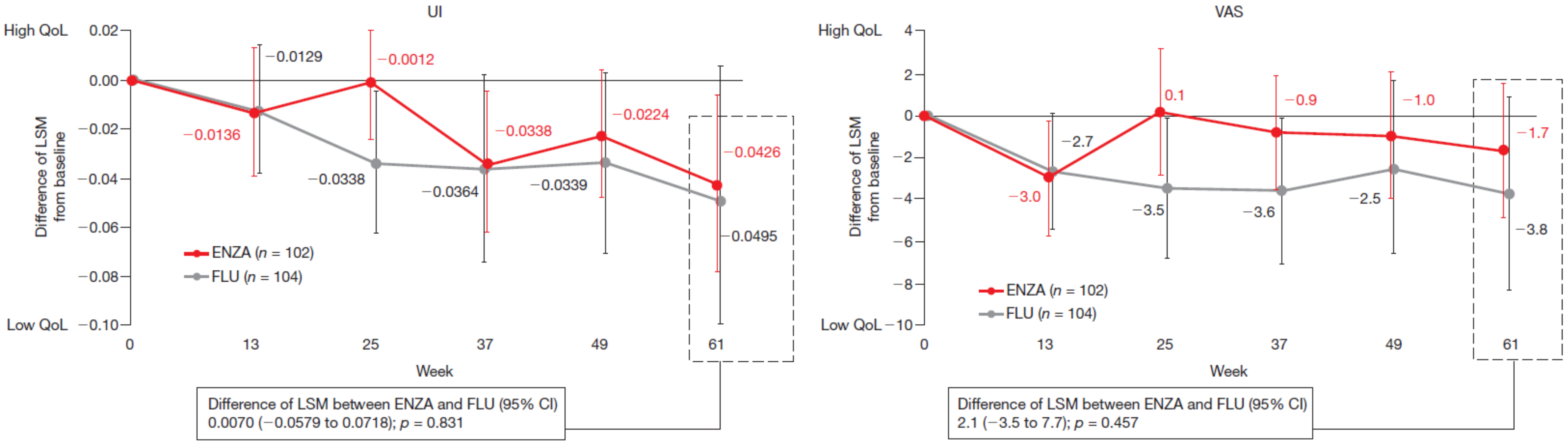

An REML approach was used. Kenward-Roger approximation was used to estimate the degree of freedom of denominator, and "unstructured" was used for structure of variance-covariance matrix

CI confidence interval; *ENZA* enzalutamide; *EQ-5D-5L* EuroQoL 5-Dimension 5-Level instruments; *FLU* flutamide; *LSM* least squares means; *QoL* quality of life; *REML* restricted maximum likelihood; *UI* utility index; *VAS* visual analog scale

Online Resource Fig. 3 Change in BPI-SF (first-line)

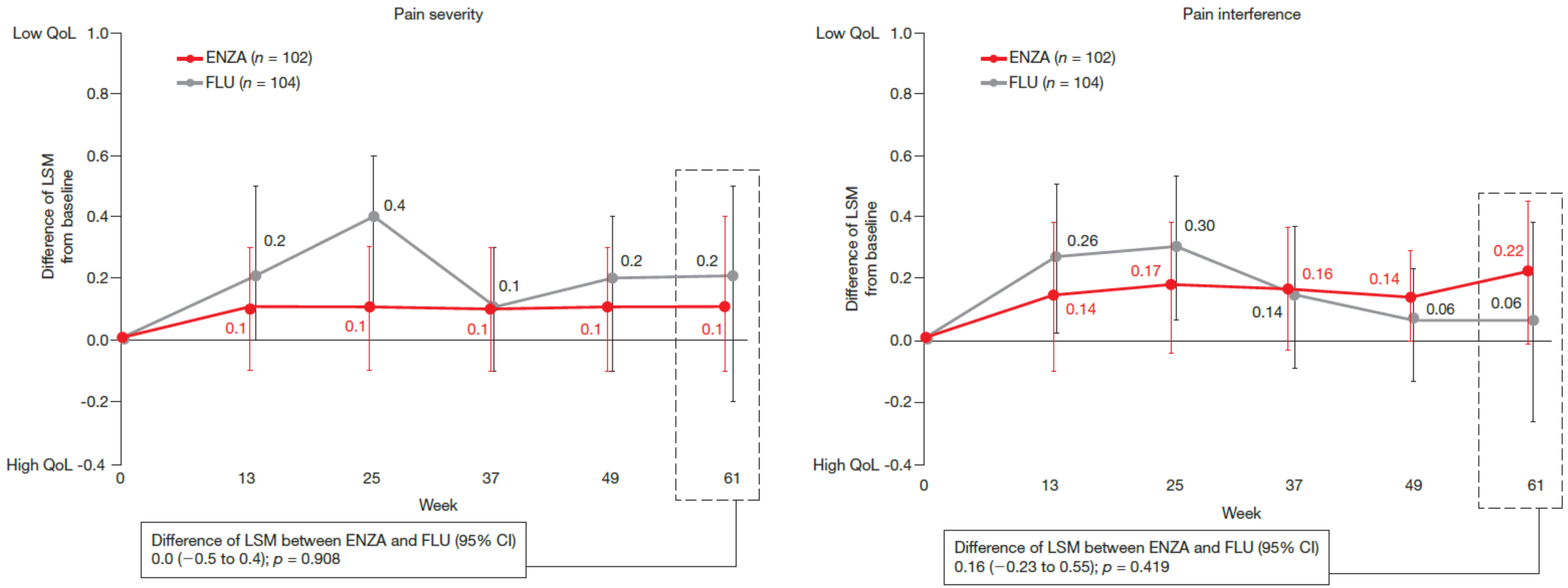

An REML approach was used. Kenward-Roger approximation was used to estimate degree of freedom of denominator, and “unstructured” was used for structure of variance-covariance matrix

BPI-SF Brief Pain Inventory–Short Form; *CI* confidence interval; *ENZA* enzalutamide; *FLU* flutamide; *LSM* least squares means; *QoL* quality of life; *REML* restricted maximum likelihood

Online Resource Fig. 4 Change in BFI global score (first-line)

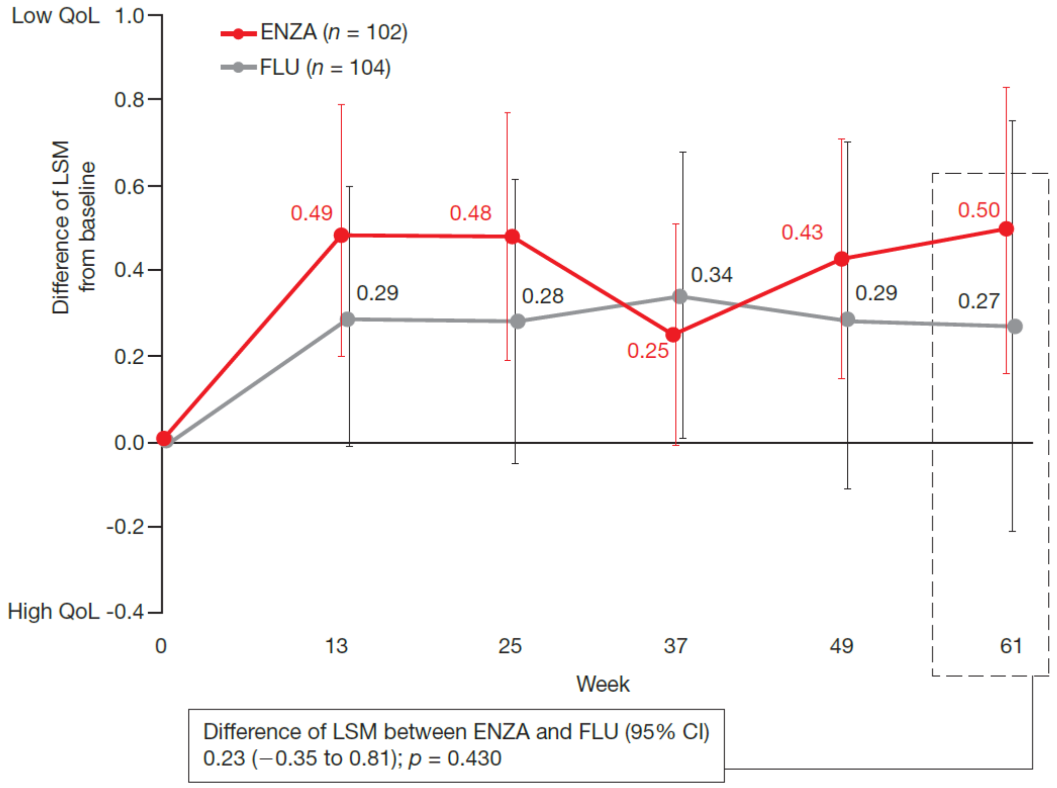

An REML approach was used. Kenward-Roger approximation was used to estimate of degree of freedom of denominator, and “unstructured” was used for structure of variance-covariance matrix

BFI Brief Fatigue Inventory; *CI* confidence interval; *ENZA* enzalutamide; *FLU* flutamide; *LSM* least squares means; *QoL* quality of life; *REML* restricted maximum likelihood
